# Colorectal cancer prevalence in faecal immunochemical test non-returners: potential for health inequality in symptomatic referral pathways

**DOI:** 10.1093/bjsopen/zrae119

**Published:** 2024-10-15

**Authors:** Adam D Gerrard, Jonty Coxon, Yasuko Maeda, Evropi Theodoratou, Malcolm G Dunlop, Farhat V N Din

**Affiliations:** Cancer Research UK Scotland Centre, Institute of Genetics and Cancer, University of Edinburgh, Edinburgh, UK; Department of Colorectal Surgery, Western General Hospital, Edinburgh, UK; Department of Colorectal Surgery, Western General Hospital, Edinburgh, UK; Cancer Research UK Scotland Centre, Institute of Genetics and Cancer, University of Edinburgh, Edinburgh, UK; Department of Surgery, Queen Elizabeth University Hospital, Glasgow, UK; Cancer Research UK Scotland Centre, Institute of Genetics and Cancer, University of Edinburgh, Edinburgh, UK; Centre for Global Health, Usher Institute, University of Edinburgh, Edinburgh, UK; Cancer Research UK Scotland Centre, Institute of Genetics and Cancer, University of Edinburgh, Edinburgh, UK; UK Colon Cancer Genetics Group, Medical Research Council Human Genetics Unit, Medical Research Council Institute of Genetics & Cancer, Western General Hospital, University of Edinburgh, Edinburgh, UK; Cancer Research UK Scotland Centre, Institute of Genetics and Cancer, University of Edinburgh, Edinburgh, UK; Department of Colorectal Surgery, Western General Hospital, Edinburgh, UK

## Abstract

**Background:**

This study aimed to describe the faecal immunochemical test non-return rate of those referred with high-risk symptoms of colorectal cancer from primary care, and the clinical outcomes of the ‘non-returners’.

**Methods:**

From January 2019 to July 2021, patients referred to secondary care with symptoms suspicious of colorectal cancer and a referral priority of urgent or urgent suspicion of cancer were sent a faecal immunochemical test. All patients were investigated regardless of faecal immunochemical test return or result. Demographics and clinical outcomes such as colorectal cancer prevalence were compared between those who returned a faecal immunochemical test and non-returners.

**Results:**

Of 7345 patients included in the study, 874 (11.9%) did not return a faecal immunochemical test. Non-returner characteristics included male sex (*P* = 0.040), younger age (median age 57 *versus* 65 years, *P* < 0.001), per rectal bleeding (*P* < 0.001) and lower socioeconomic status (median Scottish Index of Multiple Deprivation, 6 *versus* 7, *P* < 0.001) compared with those who returned a faecal immunochemical test. Of 6294 patients undergoing colorectal investigation, there was a greater prevalence of colorectal cancer (5.4% *versus* 3.6% *P* = 0.032) and significant bowel pathology than in the non-returners (15.3% *versus* 9.8%, *P* < 0.001). With a median follow-up of 25 months, the colorectal cancer prevalence for the entire 7345 cohort was equal between those who returned and did not return a faecal immunochemical test (3.2% *versus* 3.8%, *P* = 0.108). Of note, the non-returners diagnosed with colorectal cancer were younger (median age 64 *versus* 73 years, *P* < 0.001) and from a lower socioeconomic area (median Scottish Index of Multiple Deprivation 4 *versus* 7, *P* = 0.015) than faecal immunochemical test returners.

**Conclusion:**

Patients referred to secondary care, with symptoms suspicious of colorectal cancer, that did not return a faecal immunochemical test had a similar colorectal cancer prevalence to those that returned the test.

## Teaser

Faecal immunochemical testing has been adopted into clinical pathways as the first-line test to investigate people with symptoms suspicious of bowel cancer. Those who do not return faecal immunochemical tests are not well studied. We found younger males from lower socioeconomic areas were more likely to not return a faecal immunochemical test, and the rates of colorectal cancer and other significant bowel disease were higher in the non-returners, therefore, clinical pathways must reflect this.

## Introduction

Colorectal cancer (CRC) remains the third most common malignancy and second cause of cancer deaths worldwide^1^. Cancer stage determines prognosis, underscoring the importance of timely diagnosis^2^. Central to early detection are bowel screening programmes aimed at asymptomatic cases and urgent diagnostic pathways for those experiencing symptoms suggestive of colorectal cancer. The colorectal cancer yield in symptomatic patients remains around 3% despite increasing rates of referral^3,4^. The escalating pressure on services receiving referrals highlights the need to prioritize objectively patients most likely to have serious bowel pathology.

The use of faecal immunochemical testing (FIT) for haemoglobin (Hb) in the diagnostic pathway for symptomatic patients suspected to have colorectal cancer has been rapidly adopted over the past 5 years, accelerated by the endoscopy restrictions imposed during the coronavirus-19 pandemic^5^. FIT is superior to symptoms at predicting which patients have colorectal cancer and can be used to guide referral decision-making or prioritization of further investigation^6–14^.

However, the transition from a purely symptom-based referral pathway to one directed by FIT result raises the issue of management of patients that do not return their FIT. This is particularly important where FIT is used in primary care as a gatekeeper to secondary care referral. This non-returner cohort has not been well described in the emerging FIT literature, despite accounting for between 8.6% and 52.9% of eligible symptomatic patients in reported studies^7,12,15–18^.

This study aimed to describe the demographics and clinical outcomes of high-risk symptomatic patients who did not return a FIT.

## Methods

### Design and patients

Patients referred to a regional colorectal service with lower gastrointestinal symptoms and priority urgent or urgent suspicion of colorectal cancer (urgent suspicion of cancer (USoC), repeated rectal bleeding without obvious rectal cause or blood mixed in stool, persistent change in bowel habit, palpable abdominal or rectal mass, weight loss and/or abdominal pain with or without unexplained iron deficiency anaemia (IDA)) were sent either one (January 2019–February 2020) or two (March 2020–July 2021) FIT kits (Minaris Medical Co., Japan) on receipt of their referral. This formed part of routine care derived from the Scottish Government guidelines for FIT testing of patients with colorectal symptoms^19^. Upon receipt of the primary care referral, FIT kits were mailed out on the next working day and patients asked to complete the test as soon as possible and return to their general practitioner surgery. From there courier collection delivered tests to the UK Accreditation Service accredited National Health Service Tayside Blood Sciences laboratory based in Ninewells Hospital (Dundee) where, in a timely fashion, samples were analysed to International Organization for Standardization 15189 standards. Where two tests were to be sent, the second was sent upon return of the first, with a median time between tests of 13 days. Test kits included the sampling device, an information leaflet describing how to perform the test and a letter to the patient explaining that following their referral to secondary care all patients are being asked to perform a FIT that measures microscopic blood within the stool. If the kits had not been returned by day 14, a follow-up phone call was made to check that the kit had been received and to encourage return. Patients were classified as a non-returner if they did not return any FIT before investigation. The use of FIT in this referred population formed part of routine care and patients were investigated regardless of FIT return or result, with either colonoscopy or computed tomography (CT) colon protocol radiology based on their symptoms, referral information and fitness for test^5,14^.

Returned tests were processed on the HM JACK-arc analyser (Minaris Medical Co.) and a faecal haemoglobin result of 10 µg Hb/g or more was regarded as a positive test. Data was recorded prospectively. Patient demographics, referral symptoms, blood test results, and subsequent attendance and outcome from colorectal investigations was noted. Patients underwent colorectal assessment regardless of returning FIT or FIT result. Electronic patient records and cancer registries were checked to ensure complete capture of colorectal cancer including for those not attending colorectal investigation.

Due to the real-world nature of the study, it was not appropriate to delay investigations until FIT was returned. Therefore, to permit time for FIT to be completed patients who did not return a FIT but were investigated or admitted as an emergency to hospital within 7 days of referral were excluded from analysis.

### Outcomes of interest

The primary outcome of interest was to describe the demographics and clinical outcomes of high-risk symptomatic patients who did not return a FIT compared with those who returned the test.

### Statistics

Data analysis was performed using R V. 4.0.5^20^ with associated packages and GraphPad Prism^21^. Continuous data were compared by Mann–Whitney *U* test and categorical data using *X*^2^ test or Fisher's exact test as appropriate. The Scottish Index of Multiple Deprivation (SIMD) tool was used to assess levels of socioeconomic deprivation of patients within the study. This creates decile ranks from most (1) to least (10) deprived areas from postcodes. Key referral symptoms of change in bowel habit (CIBH) to looser and/or more frequent stools, per rectal (PR) bleeding, anaemia (Hb, <135 g/l in men, <120 g/l in women, as per local reference values) and the presence of an abdominal or rectal mass was recorded. Where patients had more than one symptom, all symptoms were recorded individually. The prevalence of each symptom was then calculated by dividing the absolute number by the number of patients within the cohort. The attendance of patients at planned secondary care investigations comprising of endoscopy, CT radiology or outpatient clinic appointment was collected. Non-attendance was calculated by combining occasions where patients had cancelled all future appointments or did not attend without reason. The clinical outcomes for patients who were investigated were recorded. Where a patient had more than one pathology, the most significant was used in analysis. All patients had a median follow-up of 25 months (minimum 12 months) from referral by electronic patient record and cancer registry review. No further cases of colorectal cancer were identified including in those who did not undergo initial colorectal investigations. Disease prevalence was calculated per intention analysis. A Faecal Immunochemical TesTs for Haemoglobin Evaluation Reporting checklist can be found in the *Supplementary material*. No ethical approval was required as this work formed part of routine clinical care.

## Results

### Study population

**Fig. 1:**
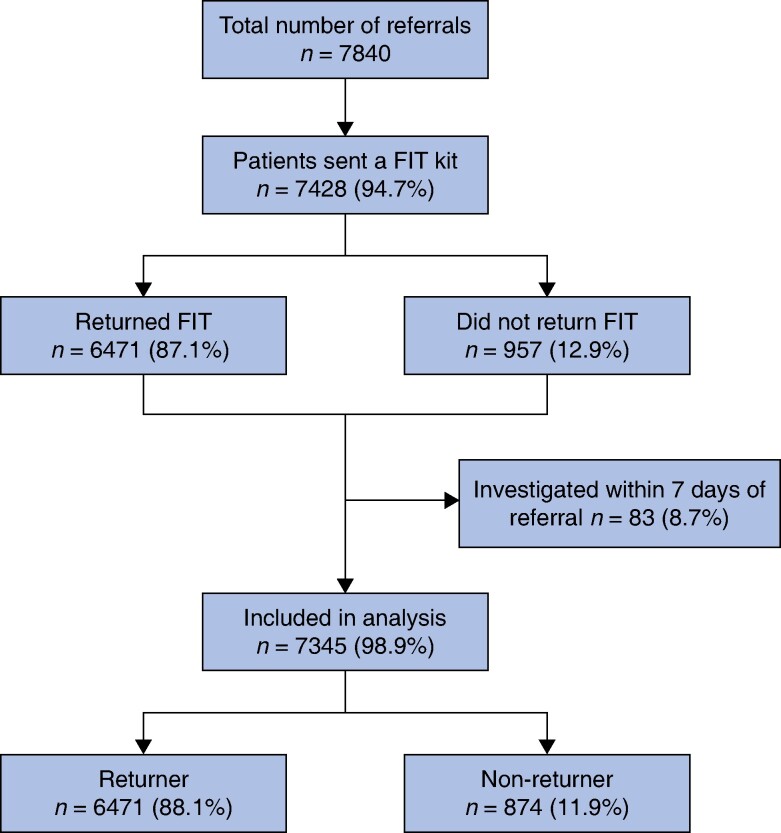
Study flow diagram FIT, faecal immunochemical testing.

A FIT kit was sent to 7428 patients (94.7%) of all urgent and USoC referrals received within the study interval. After excluding those who did not return a FIT but were investigated within 7 days of referral, 7345 patients remained for analysis (*Fig. 1*).

Eight hundred and seventy-four patients (11.9%) did not return the FIT before colorectal investigation. The non-return rate was greater at the start of the study interval and plateaued around 10% from April 2020 onwards (*Supplementary material*, *Fig. S1*). Between the two cohorts studied, the non-return rate was lower in the second cohort (March 2020–July 2021), 9.8% *versus* 14.8%, *P* < 0.0001. In those who returned a test, the positivity rate at 10 µg Hb/g was 23.6% and did not differ between the two study cohorts (*P* = 0.826). The non-returner group was younger than those who returned a FIT (*Table 1*). Those who did not return the FIT were more likely to be male, have a symptom of PR bleeding and be from a lower socioeconomic group. Upon review of the referral, a decision was made by the triaging consultant to investigate further regardless of the FIT result. In 5.9% of all referrals, it was decided from the patient record or clinic review not to investigate the colorectum; this decision did not differ between the FIT returners (5.8%) and non-returners (6.8%), *P* = 0.285. FIT non-returners triaged to investigation were then more likely to not attend these appointments compared with those offered an investigation having returned a FIT. Returners and non-returners offered colorectal investigation had similar characteristics (*Supplementary material*, *Table S1*). On electronic patient note review of the colorectal cancer registry, there had been no subsequent cases of colorectal cancer diagnosed in these patients who did not attend their initial planned investigation. Overall did not attend rate in the non-returners did not differ between the two study cohorts (January 2019–February 2020: 22.3% *versus* March 2020–July 2021: 25.2%, *P* = 0.340).

**Table 1:** Comparison of patient demographics and symptoms between those who returned and did not return a FIT

	Overall	FIT returners	FIT non-returners	*P*
No. of patients	7345	6471 (88.1)	874 (11.9)	
Age (years), median (i.q.r.)	64 (55–74)	65 (56–74)	57 (46–69)	<0.001
**Sex**				0.040
Male	3203	2794	409	
Female	4142	3677	465	
**SIMD**				
Mean(s.d.)	6.5(2.9)	6.6(2.8)	5.8(3.0)	<0.001
Median (i.q.r.)	7 (4–9)	7 (4–9)	6 (3–9)	<0.001
**Symptom prevalence**				
CIBH	4238 (56.4)	3732 (57.7)	506 (57.9)	0.901
PR bleed	2856 (38.9)	2413 (37.3)	443 (57.9)	<0.001
Anaemia	1531 (20.8)	1353 (20.9)	178 (20.4)	0.371
Abdominal mass	189 (2.6)	170 (2.6)	19 (2.2)	0.902
Rectal mass	184 (2.5)	164 (2.5)	20 (2.3)	0.730
Non-attendance for investigations	616 (8.4)	409 (6.3)	207 (23.7)	<0.001

Values are *n* (%) unless otherwise indicated. SIMD, Scottish Index of Multiple Deprivation; CIBH, Change of bowel habit to looser stool and/or diarrhoea, anaemia; Hb <135 g/l in men, <120 g/l in women, patients may report more than one symptom; FIT, faecal immunochemical testing; i.q.r., interquartile range; PR, per rectal.

### Clinical outcomes

The clinical outcomes between the two groups were then explored (*Table 2*). A total of 5686 returners and 608 non-returners underwent endoscopic or radiological investigation (median 29 days, i.q.r. 18–53, from date of referral). Within those investigated the overall prevalence of colorectal cancer was 3.8%. There was a greater rate of colorectal cancer in the FIT non-returners than returners (5.4% *versus* 3.6%, *P* = 0.032) and significant bowel pathology (colorectal cancer, advanced adenoma or inflammatory bowel disease) (15.3% *versus* 9.8%, *P* < 0.001). As all patients, including those who did not attend for colorectal investigations, had follow-up of electronic case records (median time 25 months, minimum 12 months), during which no further colorectal cancer patients were identified, the overall colorectal cancer incidence for the entire included population (*n* = 7345) can be calculated. Here, there was a 3.2% colorectal cancer prevalence with similar prevalence between the non-returners and FIT returners (3.8% *versus* 3.2%, *P* = 0.108).

**Table 2:** Pathology identified in patients who were a FIT returner *versus* non-returner and subsequently underwent colorectal investigation (*n* = 6294)

	FIT returners	FIT non-returners	*P*
No. of patients	5686	608	
**Pathology**			
Colorectal cancer	204 (3.6)	33 (5.4)	0.032
Advanced adenoma	240 (4.2)	33 (5.4)	0.173
Advanced colorectal neoplasia	444 (7.8)	66 (10.9)	0.012
Inflammatory bowel disease	113 (2.0)	27 (4.4)	<0.001
Significant bowel pathology	557 (9.8)	93 (15.3)	<0.001

Values are *n* (%) unless otherwise indicated. Advanced colorectal neoplasia (colorectal cancer + advanced adenoma); significant bowel pathology (advanced colorectal neoplasia + inflammatory bowel disease). FIT, faecal immunochemical testing.

Exploring these cases of colorectal cancer further, there was no difference in sex distribution between the returners and non-returners who developed cancer (*Table 3*). Those who did not return a FIT and were subsequently diagnosed with colorectal cancer were younger and of lower socioeconomic status than the FIT returners. There was no difference in cancer location within the colorectum, or AJCC stage where the colorectal cancer was resected; however, more patients who did not return a FIT were palliated compared with those who did (9.1% *versus* 1.5%, *P* = 0.036).

**Table 3:** Patient demographic, clinical and pathology features of colorectal cancer diagnosed in FIT returners and non-returners (*n* = 7345)

	FIT returners	FIT non-returners	*P*
No. CRC	204	33	
Age (years), median (i.q.r.)	73 (64–80)	64 (53–73)	<0.001
**Sex**			0.710
Male	103	18	
Female	101	15	
**SIMD**			
Mean(s.d.)	6.8(2.8)	5.3(3.0)	0.009
Median (i.q.r.)	7 (4–10)	4 (3–7)	0.015
**CRC location**			
Right	57 (27.9)	7 (21.2)	0.528
Left	45 (22.1)	9 (27.3)	0.507
Rectal	102 (50.0)	17 (51.5)	0.999
**AJCC stage**			
1	28 (13.7)	5 (15.2)	0.789
2	60 (29.4)	8 (24.2)	0.679
3	64 (31.4)	9 (27.3)	0.690
4	49 (24.0)	8 (24.2)	0.999
Palliated (not resected)	3 (1.5)	3 (9.1)	0.036

Values are *n* (%) unless otherwise indicated. SIMD, Scottish Index of Multiple Deprivation; CRC location right; caecum to before splenic flexure, left; splenic flexure to rectosigmoid, rectal; rectosigmoid and rectum. AJCC, American Joint Committee on Cancer staging; FIT, faecal immunochemical testing; CRC, colorectal cancer; i.q.r., interquartile range.

## Discussion

This study estimates the FIT non-return rate in symptomatic patients with an urgent or urgent suspicion of cancer priority referral from primary care. Those not to return a FIT were more likely to be male, younger, from a lower socioeconomic area and to have a symptom of rectal bleeding. There was at least equal prevalence of colorectal cancer between those who returned and did not return a FIT, with more significant bowel pathology in the latter. Within the cases of colorectal cancer, the non-returners continued to be younger and from lower socioeconomic areas.

The non-return rate of FIT in this study was 11.9%. An improvement was observed in the return rate in the second cohort of the study (March 2020 to July 2021) and particularly after April 2020, from which the non-return rate was steady at around 10%. This improvement may have been influenced by the COVID-19 pandemic, with patients being referred that were potentially more symptomatic or with increased health-seeking behaviour at that time. However, the compliance with FIT continued into the following year post-COVID-19, suggesting that in fact the improvement was more wide-reaching than the effect of the pandemic. It likely involved multiple components, including increased awareness of FIT in primary care resulting in early discussions with patients to expect the test and improvements from growing experience within the secondary care service. Assessing the real-world non-return rate of FIT in symptomatic patients is difficult due to varying study designs, methodology and populations studied. In the UK, where FIT has been used in clinical practice, the non-return rate varies from 8.6% to 52.9%^7,12,15–18,22^. The lower rate of 8.6% occurred where patients with rectal bleeding were excluded; as shown in this study this subgroup accounted for significantly more non-returners and so may explain this lower non-return rate^12^. The highest reported non-return rate (52.9%) occurred in Scotland where FIT was organized in general practice alongside referral to secondary care^18^. This was similar to the uptake of guaiac-based bowel screening in Scotland at this time (55.4%). Subsequently as FIT was introduced to the Scottish Bowel Screening Programme, the return rate increased to 67%, but a wide disparity existed between those in the least (75%) and most (54%) deprived areas, mirroring what we observed symptomatically^23^. A further recent real-world study of FIT in symptomatic referrals from England reported that when required from primary care alongside referral, 52% of patients did not complete a FIT^22^. This poor compliance with FIT completion highlights the significant potential impact on primary care workload, especially where a FIT result is requested for review on urgent cancer pathways, to follow-up patients to ensure the tests were complete. The Association of Coloproctology of Great Britain and Ireland (ACPGBI) and the British Society of Gastroenterology (BSG) guidelines for the use of FIT in patients with signs and symptoms suggestive of colorectal cancer, published after this study, advise FIT testing in primary care before referral to secondary care service by urgent suspected cancer pathways^24^. Whilst there is safety netting and non-urgent pathways available for either negative FIT results or potentially FIT non-returners, these will inevitably come with delays to diagnosis. As demonstrated by the studies above, compliance with FIT in primary care is substantially lower than we have described using a dedicated secondary care pathway, and as such, regional routine clinical practice has been to retain FIT in secondary care. The colorectal cancer prevalence in this study of the FIT returners was the same as in patients who did return the test, therefore non-returners cannot be safely left without follow-up.

In fact the prevalence of colorectal cancer in the non-returners within this study (3.8%) is comparable with the reported prevalence in the literature of symptomatic patients who return FIT tests with results between 20–49 µg Hb/g (3.6%)^10^ and 10–99.9 µg Hb/g (3.8%)^6^.

Generally, FIT is acceptable to patients with high levels of test satisfaction reported^25^. Reasons for FIT non-return in symptomatic patients are not well studied. Equity of access to secondary care services should be ensured for symptomatic patients who do not return a FIT. Screening data suggest compliance is improved when patients receive prior notification of the FIT^26^ and when reminders are sent to those where test returns are awaited^27,28^. It was previously shown the benefit of telephone contact when FIT was being used during a COVID-adapted pathway^29^. Proactive telephone contact may explain the improved non-return rate in our study compared with similar FIT studies where kits were mailed out without follow-up. On the other hand, in this study, clinical pathways were not delayed for FIT return. Not delaying investigations would have created a higher non-return rate compared with a setting where no investigations were ordered until a FIT was returned, however, due to the real-world setting of the study this was not feasible.

This large prospective study with near total capture of the referred population has allowed an accurate analysis of the non-return rate of FIT in a high-risk symptomatic population and described the clinical outcomes of this group. The study is limited in that data of patient ethnicity was unavailable; however, given Scotland has a population comprising of 94.9% of White Caucasian origin, no conclusions would have been valid^30^. Ethnic inequalities are reported within the bowel screening programme in other areas of the UK^31^. Ethnicity is a potential health inequality in FIT-directed pathways and should be considered in service planning and outreach programmes. Furthermore, this study was also conducted in secondary care in patients who had been referred due to their symptoms. Therefore, the results may not be generalizable to a setting where FIT is performed on all primary care attendees with bowel symptoms. This study did not aim to understand why patients did not complete their FIT, and future qualitative work will be essential to pathway design.

There is a high non-attendance rate for further investigation in those who did not return a FIT (23.7%). Hence, it may seem appealing given healthcare resources are limited not to offer further investigations unless a FIT is returned. However, whilst no colorectal cancer was identified from colorectal cancer registries in those non-returners who did not attend planned investigation, there was significant bowel pathology including colorectal cancer in the non-returners who did attend investigation. Reasons for non-attending investigations were not part of the remit of this study, but given the colorectal cancer rate in the FIT non-returners who were investigated, caution must be exercised against a default approach of non-investigation in the case of FIT non-return.

When used in primary care for the assessment of colorectal symptoms, uptake of FIT has been shown to be lower in men, people under 65 years, those from lower socioeconomic backgrounds and of non-White ethnicity^32^. Colorectal cancer rates were lower in the non-returners (1.0% *versus* 1.6%) than the returners in this study from primary care. In comparison, the present study has found similar demographic trends in those symptomatic patients who participate in FIT testing. However, a strength of this investigation is that all patients were offered investigations if appropriate regardless of FIT, or any other serum marker. The colorectal cancer prevalence was also higher in our study suggesting that the population being studied has more high-risk symptomatic patients, or there are cancers yet to be diagnosed in the comparative study.

The finding of equal colorectal cancer prevalence is critical to the service development of FIT-directed referral pathways for suspected colorectal cancer. Non-returners in general, and those with colorectal cancer, were younger and from lower socioeconomic areas, and risk being disadvantaged if FIT is the gatekeeper to investigation of symptoms. Indeed, younger patients with colorectal cancer already face delayed times to diagnosis and often a worse stage cancer^33,34^. Socioeconomic deprivation is linked with reduced participation in bowel screening, increased risk of colorectal cancer development, later diagnosis and worse outcomes^35–38^. FIT-directed pathways must account for means to assess the individual risk of non-returners. This work highlights the potential danger of implementing a FIT-directed strategy without due regard for all groups within the population.

## Supplementary Material

zrae119_Supplementary_Data

## Data Availability

Summarized anonymized data will be made available on request.

## References

[1] Xi Y, Xu P. Global colorectal cancer burden in 2020 and projections to 2040. Transl Oncol 2021;14:101174 34243011 10.1016/j.tranon.2021.101174 PMC8273208

[2] Siegel RL, Miller KD, Fedewa SA, Ahnen DJ, Meester RGS, Barzi A et al Colorectal cancer statistics, 2017. CA Cancer J Clin 2017;67:177–193 28248415 10.3322/caac.21395

[3] Maclean W, Singh R, Mackenzie P, White D, Benton S, Stebbing J et al The two-week rule colorectal cancer pathway: an update on recent practice, the unsustainable burden on diagnostics and the role of faecal immunochemical testing. Ann R Coll Surg Engl 2020;102:308–311 32081023 10.1308/rcsann.2020.0019 PMC7099154

[4] NICE . NICE Guideline NG12: Suspected Cancer: Recognition and Referral. National Institute for Health and Care Excellence, 2015. https://www.nice.org.uk/guidance/conditions-and-diseases/cancer/colorectal-cancer (accessed 11 March 2024) 26180880

[5] Miller J, Thomson LJ, Stewart LSP, Fleming J, Dunlop MG, Din FVN et al Implementation of a risk mitigating COVID-adapted colorectal cancer pathway. BMJ Open Qual 2021;10:e001135 10.1136/bmjoq-2020-001135 PMC7802393 33431430

[6] Bailey JA, Ibrahim H, Bunce J, Chapman CJ, Morling JR, Simpson JA et al Quantitative FIT stratification is superior to NICE referral criteria NG12 in a high-risk colorectal cancer population. Tech Coloproctol 2021;25:1151–1154 34263362 10.1007/s10151-021-02466-z PMC8279105

[7] D’Souza N, Georgiou Delisle T, Chen M, Benton S, Abulafi M; NICE FIT Steering Group. Faecal immunochemical test is superior to symptoms in predicting pathology in patients with suspected colorectal cancer symptoms referred on a 2WW pathway: a diagnostic accuracy study. Gut 2021;70:1130–1138 33087488 10.1136/gutjnl-2020-321956 PMC8108285

[8] Cubiella J, Salve M, Díaz-Ondina M, Vega P, Alves MT, Iglesias F et al Diagnostic accuracy of the faecal immunochemical test for colorectal cancer in symptomatic patients: comparison with NICE and SIGN referral criteria. Int J Colorectal Dis 2014;16:O273–O282 10.1111/codi.12569 24456168

[9] Mowat C, Digby J, Strachan JA, McCann RK, Carey FA, Fraser CG et al Faecal haemoglobin concentration thresholds for reassurance and urgent investigation for colorectal cancer based on a faecal immunochemical test in symptomatic patients in primary care. Ann Clin Biochem 2021;58:211–219 33334134 10.1177/0004563220985547 PMC8114428

[10] McSorley ST, Digby J, Clyde D, Cruickshank N, Burton P, Barker L et al Yield of colorectal cancer at colonoscopy according to faecal haemoglobin concentration in symptomatic patients referred from primary care. Colorectal Dis 2021;23:1615–1621 33064898 10.1111/codi.15405

[11] Johnstone MS, Burton P, Kourounis G, Winter J, Crighton E, Mansouri D et al Combining the quantitative faecal immunochemical test and full blood count reliably rules out colorectal cancer in a symptomatic patient referral pathway. Int J Colorectal Dis 2022;37:457–466 34932152 10.1007/s00384-021-04079-2 PMC8803704

[12] Chapman C, Thomas C, Morling J, Tangri A, Oliver S, Simpson JA et al Early clinical outcomes of a rapid colorectal cancer diagnosis pathway using faecal immunochemical testing in Nottingham. Colorectal Dis 2020;22:679–688 31876975 10.1111/codi.14944

[13] D’Souza N, Delisle TG, Chen M, Benton SC, Abulafi M; NICE FIT Steering Committee. Faecal immunochemical testing in symptomatic patients to prioritize investigation: diagnostic accuracy from NICE FIT study. Br J Surg 2021;108:804–810 33755051 10.1093/bjs/znaa132

[14] Gerrard AD, Maeda Y, Miller J, Gunn F, Theodoratou E, Noble C et al Double faecal immunochemical testing in patients with symptoms suspicious of colorectal cancer. Br J Surg 2023;110:471–480 36785496 10.1093/bjs/znad016 PMC10364540

[15] Maclean W, Mackenzie P, Limb C, Zahoor Z, Whyte MB, Rockall T et al Diagnostic accuracy of point of care faecal immunochemical testing using a portable high-speed quantitative analyser for diagnosis in 2-week wait patients. Colorectal Dis 2021;23:2376–2386 34157205 10.1111/codi.15780

[16] Widlak MM, Thomas CL, Thomas MG, Tomkins C, Smith S, O’Connell N et al Diagnostic accuracy of faecal biomarkers in detecting colorectal cancer and adenoma in symptomatic patients. Aliment Pharmacol Ther 2017;45:354–363 27910113 10.1111/apt.13865

[17] Turvill JL, Turnock D, Cottingham D, Haritakis M, Jeffery L, Girdwood A et al The fast track FIT study: diagnostic accuracy of faecal immunochemical test for haemoglobin in patients with suspected colorectal cancer. Br J Gen Pract 2021;71:e643–e651 33798091 10.3399/BJGP.2020.1098 PMC8279659

[18] Mowat C, Digby J, Strachan JA, Wilson R, Carey FA, Fraser CG et al Faecal haemoglobin and faecal calprotectin as indicators of bowel disease in patients presenting to primary care with bowel symptoms. Gut 2016;65:1463–1469 26294695 10.1136/gutjnl-2015-309579 PMC5036251

[19] Scottish Government . FIT Testing for Patients With Colorectal Symptoms: Primary Care Guidance. 2022. https://www.gov.scot/publications/primary-care-guidance-use-fit-testing-patients-colorectal-symptoms/ (accessed 11 March 2024)

[20] R Core Team . R: A Language and Environment for Statistical Computing. Vienna, Austria: R Foundation for Statistical Computing, 2013. http://www.R-project.org/

[21] GraphPad . *Prism Version 8.0.0 for Windows GS*, San Diego, California, USA. www.graphpad.com (accessed 11 March 2024)

[22] Rahman F, Trivedy M, Rao C, Akinlade F, Mansuri A, Aggarwal A et al Faecal immunochemical testing to detect colorectal cancer in symptomatic patients: a diagnostic accuracy study. Diagnostics (Basel) 2023;13:2332 37510076 10.3390/diagnostics13142332 PMC10378039

[23] Public Health Scotland . *Scottish Bowel Screening Programme Statistics: For the Period of Invitations from May 2020 to April 2022*. 2023. https://publichealthscotland.scot/publications/scottish-bowel-screening-programme-statistics/scottish-bowel-screening-programme-statistics-for-the-period-of-invitations-from-may-2020-to-april-2022/ (accessed 10 March 2024)

[24] Monahan KJ, Davies MM, Abulafi M, Banerjea A, Nicholson BD, Arasaradnam R et al Faecal immunochemical testing (FIT) in patients with signs or symptoms of suspected colorectal cancer (CRC): a joint guideline from the Association of Coloproctology of Great Britain and Ireland (ACPGBI) and the British Society of Gastroenterology (BSG). Gut 2022;71:1939–1962 35820780 10.1136/gutjnl-2022-327985 PMC9484376

[25] Gil N, Su H, Kaur K, Barnett M, Murray A, Duffy S et al Patient experience and satisfaction with symptomatic faecal immunochemical testing: an explanatory sequential mixed-methods evaluation. Br J Gen Pract 2023;73:e104–e114 36702594 10.3399/BJGP.2022.0241 PMC9888563

[26] van Roon AH, Hol L, Wilschut JA, Reijerink JC, van Vuuren AJ, van Ballegooijen M et al Advance notification letters increase adherence in colorectal cancer screening: a population-based randomized trial. Prev Med 2011;52:448–451 21457725 10.1016/j.ypmed.2011.01.032

[27] Piette C, Durand G, Bretagne JF, Faivre J. Additional mailing phase for FIT after a medical offer phase: the best way to improve compliance with colorectal cancer screening in France. Dig Liver Dis 2017;49:308–311 27810401 10.1016/j.dld.2016.09.015

[28] Fujita M, Fujisawa T, Hata A. Additional outreach effort of providing an opportunity to obtain a kit for fecal immunochemical test during the general health check-up to improve colorectal cancer screening rate in Japan: a longitudinal study. PLoS One 2020;15:e0238474 32866208 10.1371/journal.pone.0238474 PMC7458287

[29] Bell SE, Crawford J, Gunn F, Noble C, Miller J, Dunlop MG et al Nurse-led telephone outreach for a COVID-adapted suspected colorectal cancer pathway. Gastrointest Nurs 2021;19:22–26

[30] House of Commons Library . *Ethnic Diversity in Politics and Public Life*. 2021. https://commonslibrary.parliament.uk/research-briefings/sn01156/

[31] Sekhon Inderjit Singh HK, Lal N, Majeed A, Pawa N. Ethnic disparities in the uptake of colorectal cancer screening: an analysis of the west London population. Colorectal Dis 2021;23:1804–1813 33880876 10.1111/codi.15682

[32] Bailey JA, Morton AJ, Jones J, Chapman CJ, Oliver S, Morling JR et al Sociodemographic variations in the uptake of faecal immunochemical tests in primary care: a retrospective study. Br J Gen Pract 2023;73:e843–e849 37845084 10.3399/BJGP.2023.0033 PMC10587902

[33] Taggarshe D, Rehil N, Sharma S, Flynn JC, Damadi A. Colorectal cancer: are the “young” being overlooked? Am J Surg 2013;205:312–316; discussion 316 23414955 10.1016/j.amjsurg.2012.10.016

[34] Araujo L, Breau G, George M, Dau H, Gastonguay L, Brown EH et al Shared experiences of diagnosis and treatment of young-onset colorectal cancer: a patient-oriented qualitative study. J Psychosoc Oncol Res Pract 2020;2:e17

[35] Mosquera I, Mendizabal N, Martín U, Bacigalupe A, Aldasoro E, Portillo I; from the Desberdinak Group. Inequalities in participation in colorectal cancer screening programmes: a systematic review. Eur J Public Health 2020;30:558–567 10.1093/eurpub/ckz236 32361732

[36] Doubeni CA, Laiyemo AO, Major JM, Schootman M, Lian M, Park Y et al Socioeconomic status and the risk of colorectal cancer. Cancer 2012;118:3636–3644 22898918 10.1002/cncr.26677 PMC3422782

[37] Patel A, Gantz O, Zagadailov P, Merchant AM. The role of socioeconomic disparity in colorectal cancer stage at presentation. Updates Surg 2019;71:523–531 30788664 10.1007/s13304-019-00632-5

[38] van den Berg I, Buettner S, van den Braak RRJC, Ultee KHJ, Lingsma HF, van Vugt JLA et al Low socioeconomic status is associated with worse outcomes after curative surgery for colorectal cancer: results from a large, multicenter study. J Gastrointest Surg 2020;24:2628–2636 31745899 10.1007/s11605-019-04435-2 PMC7595960

