# Supplementary material for: Colorectal cancer prevalence in faecal immunochemical test non-returners: potential for health inequality in symptomatic referral pathways
Source: BJS Open. 2024 Oct 15;8(5):zrae119. doi: 10.1093/bjsopen/zrae119 (PMC11474236; doi:10.1093/bjsopen/zrae119)
Supplement: zrae119_Supplementary_Data [file zrae119_supplementary_data.docx]

**Title:**

Colorectal cancer prevalence in faecal immunochemical test non-returners: potential for health inequality in symptomatic referral pathways

**Authors:**

Gerrard AD^1,2^, Coxon J^2^, Maeda Y^1,3^, Theodoratou E^1,4^, Dunlop MG^1,5^, Din FVN^1,2^

^1^ *Cancer Research UK Scotland Centre, Institute of Genetics and Cancer, University of Edinburgh, Edinburgh, UK*

^2^ *Department of Colorectal Surgery, Western General Hospital, Edinburgh, Scotland*

^3^ *Department of Surgery, Queen Elizabeth University Hospital, Glasgow, Scotland*

^4^ *Centre for Global Health, Usher Institute, The University of Edinburgh, Edinburgh, Scotland*

^5^ *UK Colon Cancer Genetics Group, Medical Research Council Human Genetics Unit, Medical Research Council Institute of Genetics & Cancer, Western General Hospital, The University of Edinburgh, Edinburgh, UK*

**Corresponding Author**

Farhat Din Reader & Honorary Consultant Colorectal Surgeon

Address: Cancer Research UK Scotland Centre, Institute of Genetics and Cancer, University of Edinburgh, Crewe Road, Edinburgh, UK, EH4 2XU

Email: [Farhat.Din@ed.ac.uk](mailto:Farhat.Din@ed.ac.uk)

**Supplementary Materials Index**

FITTER checklist pag.2

Figure 1:

Non-return rates over time pag. 3

Table 1:

Comparison of returners and non-returners who did not attend colorectal investigation pag. 4

**Supplementary File 1: FITTER Checklist**

*Specimen collection and handling*

In cohort 1 (January 2019 – February 2020) patients referred to secondary care (3074) were sent one specimen collection device (Minaris Medical Co. Ltd) and cohort 2 (March 2020 – July 2021) patients (4354) were sent two collection devices on average 13 days apart. The device collects 2mg of faeces with a probe attached to the cap into 2.0ml of buffer. Participants pass the probe into the stool, wrote the date of collection, and returned the collection kits to their local primary care centre who transported the kits to the regional laboratory (Dundee, Scotland) where the UKAS accredited NHS Tayside Blood Sciences laboratory is based in Ninewells Hospital. In timely fashion samples were analysed to ISO15189 standards. In total, 6471 patients completed at least one FIT.

*Analysis*

Specimens were analysed using the HM-JACKarc analyser. Samples were stored at 4°C until the time of analysis and allowed to warm to room temperature. The analytical working range in 7-400µg Hb/g.

*Quality management*

All analysis is carried out at the regional laboratory. There are daily internal quality control measures with repeated sampling and between batch impression aiming for ±2SD. External quality assessment is performed three times per month with sample material prepared by EQU laboratories (Birmingham, England).

*Data Handling*

The f-Hb concentrations are recorded electronically and linked back to the patient record to be available to the clinical team.

Supplementary Figure 1: Non-return rates over time

Supplementary Table 1: Comparison of returners and non-returners who did not attend colorectal investigation

|  | **Returner** | **Non-returner** | ***p-value*** |
| --- | --- | --- | --- |
| **Number of Patients *(%)*** | 409 | 207 |  |
| **Age, years - median - (IQR)** | 64 (55-74) | 57 (46-70) | *<0.001* |
| **Sex, F *(%)*** | 245 *(59.9)* | 102 *(50.7)* | *0.030* |
| **SIMD** |  |  |  |
| Mean (SD) | 6.0 (3.0) | 5.6 (3.0) | *0.081* |
| Median (IQR) | 6 (3-9) | 5 (3-8) | *0.084* |
| **Symptom Prevalence *(%)*** |  |  |  |
| CIBH | 222 *(54.3)* | 113 *(54.6)* | *0.999* |
| PR Bleed | 155 *(37.9)* | 84 *(40.6)* | *0.541* |
| Anaemia | 91 *(22.2)* | 49 *(23.7)* | *0.685* |
| Abdominal Mass | 6 *(1.5)* | 4 *(1.9)* | *0.739* |
| Rectal Mass | 7 *(1.7)* | 1 *(0.5)* | *0.278* |

SIMD: Scottish Index of Multiple Deprivation; CIBH; Change of bowel habit to looser stool and/or diarrhoea, Anaemia; Hb <135g/L in males, <120g/L in females, Patients may report more than one symptom.
